# The Synthetic Cannabinoids THJ-2201 and 5F-PB22 Enhance In Vitro CB_1_ Receptor-Mediated Neuronal Differentiation at Biologically Relevant Concentrations

**DOI:** 10.3390/ijms21176277

**Published:** 2020-08-30

**Authors:** João Alexandre, Rui Malheiro, Diana Dias da Silva, Helena Carmo, Félix Carvalho, João Pedro Silva

**Affiliations:** UCIBIO, REQUIMTE, Laboratory of Toxicology, Department of Biological Sciences, Faculty of Pharmacy, University of Porto, 4050-313 Porto, Portugal; jalexndr1995@hotmail.com (J.A.); rui_malheiro@outlook.com (R.M.); dsilva@ff.up.pt (D.D.d.S.); helenacarmo@ff.up.pt (H.C.)

**Keywords:** developmental neurotoxicity, endocannabinoid system, neurogenesis, new psychoactive substances, substances of abuse

## Abstract

Recreational use of synthetic cannabinoids (SCs) before and during pregnancy poses a major public health risk, due to the potential onset of neurodevelopmental disorders in the offspring. Herein, we report the assessment of the neurotoxic potential of two commonly abused SCs, THJ-2201 and 5F-PB22, particularly focusing on how they affect neuronal differentiation in vitro. Differentiation ratios, total neurite length, and neuronal marker expression were assessed in NG108-15 neuroblastoma x glioma cells exposed to the SCs at non-toxic, biologically relevant concentrations (≤1 μM), either in acute or repeated exposure settings. Both SCs enhanced differentiation ratios and total neurite length of NG108-15 cells near two-fold compared to vehicle-treated cells, in a CB_1_R activation-dependent way, as the CB_1_R blockade with a specific antagonist (SR141718) abrogated SC-induced effects. Interestingly, repeated 5F-PB22 exposure was required to reach effects similar to a single THJ-2201 dose. Cell viability and proliferation, mitochondrial membrane potential, and intracellular ATP levels were also determined. The tested SCs increased mitochondrial tetramethyl rhodamine ethyl ester (TMRE) accumulation after 24 h at biologically relevant concentrations but did not affect any of the other toxicological parameters. Overall, we report firsthand the CB_1_R-mediated enhancement of neurodifferentiation by 5F-PB22 and THJ-2201 at biologically relevant concentrations.

## 1. Introduction

Synthetic cannabinoids (SCs) comprise a chemically heterogenous group of new psychoactive substances, representing the largest group of new psychoactive substances monitored by the European Monitoring Centre for Drugs and Drug Addiction (EMCDDA), and accounting for 51% of total seizures of new psychoactive substances in the European Union [1]. They are structurally designed to mimic, but with higher potency, the action of tetrahydrocannabinol (THC, the main psychoactive component of cannabis), by targeting the endocannabinoid (eCB) system via the activation of at least one of the main cannabinoid receptors (CBRs): CB_1_R and CB_2_R. While phytocannabinoids are typically partial agonists of CBRs, SCs are full agonists of these receptors, accounting for their stronger psychoactive, as well as other adverse effects [2]. Rising SC-related intoxications and deaths embody a major challenge for public health and policy-makers [1]. Most important, the recent changes in cannabis and its derivatives’ legal status may likely lead to an increased use of more potent cannabinoids, as these become cheaper and more readily available [3]. As such, the number of reports on SC-linked emergency room visits may aggravate in the near future. Pregnant and women of childbearing age comprise specific risk groups. In fact, the use of cannabis during pregnancy has considerably increased over the past decade (prevalence rate of 3–16% in western societies), mostly as a self-medication for nausea and vomiting [4,5]. Prenatal cannabis exposure is associated with a high risk of adverse maternal and neonatal outcomes, including neuronal development impairment that may trigger permanent behavioral effects (e.g., schizophrenia, autism spectrum disorders, anxiety, and mood disorders) in the offspring, which can be detected in early infancy or throughout child development [4,6,7].

Similarly to THC and other phytocannabinoids, SCs interfere with the eCB system-mediated signaling, which tightly controls neurodevelopment-related processes in the developing brain, including neuronal proliferation, differentiation, synapse formation, and pruning [4,8]. However, to the best of our knowledge, the neurobiology underlying the impact of SCs’ use by pregnant women and women of childbearing age on their offspring remains virtually unexplored, with only a few studies correlating impaired cognition with perinatal SC exposure, as we previously reviewed [9]. Of note, these data are often conflicting, varying among cell models or types of SCs used. For example, Kim and Thayer [10] observed that WIN55,212-2 blocked the formation of new synapses in cultured hippocampal neurons. Mereu et al. showed that exposure of pregnant rats to a non-toxic dose of WIN55, 212-2 led to memory impairment in 40- and 80-day-old offspring [11]. Psychoyos et al. observed abnormal plate formation and neural tube closing in chicks following embryo exposure to the SC O-2545 [12]. Enhanced proliferation, but not differentiation, of neural stem [13] or progenitor cells [14] was reported after exposure to HU-210.

We hypothesize that SCs may be neurotoxic, possibly by modulating neurodevelopment-related processes. The analysis of SCs neurotoxic potential assumes critical relevance to strengthen SC risk assessment, especially by contributing to elucidate the impact of these substances’ use by pregnant women and women of childbearing age. In this sense, this work comprises two main goals: (1) to evaluate the neurotoxicity of two commonly reported SCs—THJ-2201 and 5F-PB22—in a NG108-15 hybrid neuroblastoma x glioma cell line; (2) to assess the role played by these SCs on in vitro neuronal differentiation and proliferation of NG108-15 cells exposed at non-toxic, biologically relevant concentrations (<1 μM), in acute and repeated exposure settings. The potential involvement of the CB_1_R in these SC-mediated processes is assessed in the presence of a specific CB_1_R antagonist. Of note, the NG108-15 cell line represents a well-characterized model of neuritogenesis from a blastoid state under stressed conditions, commonly used to study neuronal development and differentiation [15]. Differentiated NG108-15 cells exhibit well-characterized morphological properties similar to neurons, including neurite outgrowth and a synchronized differentiation, which allows the examination of morphological changes triggered by SCs [15,16,17].

## 2. Results

### 2.1. Cell Viability and Proliferation Were Not Affected by 5F-PB22 or THJ-2201 at Biologically Relevant Concentrations

**Figure 1:**
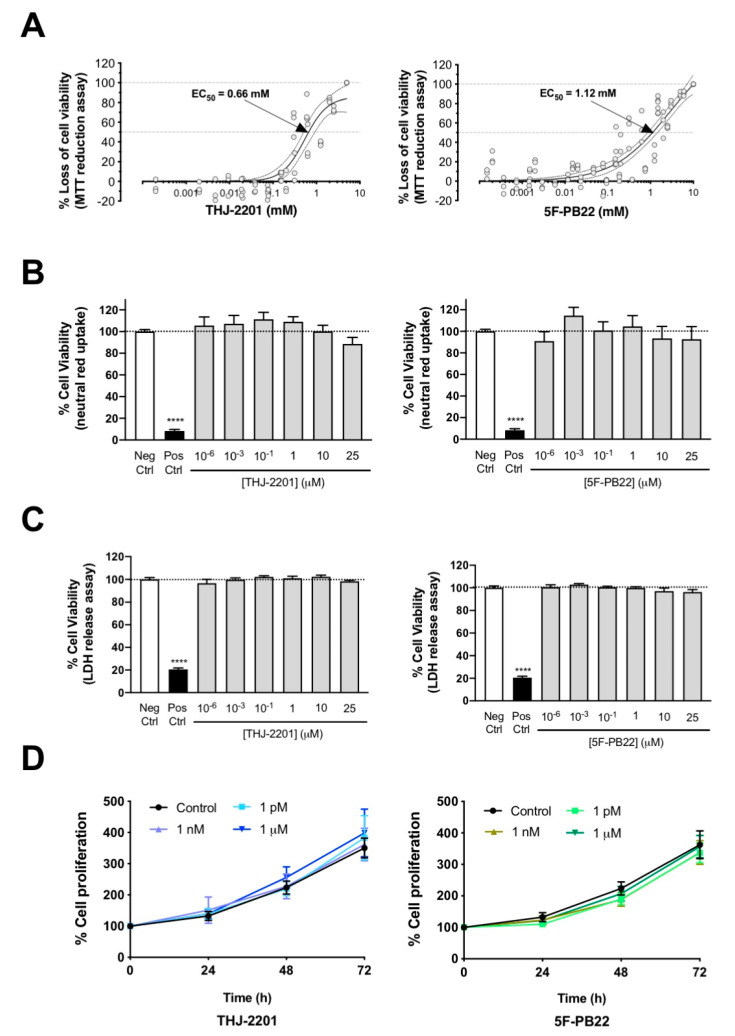
Cell viability and proliferation assessed in NG108-15 cells exposed to either THJ-2201 or 5F-PB22. Distinct biomarkers of cell viability were determined after 24 h-exposure at a range of drug concentrations described in Materials and Methods. (**A**) Metabolic activity, assessed by the 3-(4,5-dimethylthiazol-2-yl)-2,5-diphenyltetrazolium (MTT) reduction assay. Each concentration was tested in triplicate in four independent experiments. EC_50_ is the drug concentration that induces half-maximal response (i.e., 50% effect); (**B**) Lysosomal integrity, analyzed by the neutral red uptake assay. Each bar represents the mean ± SEM, for at least six independent experiments, performed in duplicate. **** *p* < 0.0001, compared to the control (0.1% DMSO), using an unpaired, two-tailed *t*-test. No statistically significant differences were observed for any of the SC concentrations tested, using an one-way ANOVA, followed by Dunnett’s post-test; (**C**) Membrane integrity, evaluated by the lactate dehydrogenase (LDH) release assay. Each bar represents the mean ± SEM, for at least six independent experiments, performed in duplicate. **** *p* < 0.0001, compared to the control (0.1% DMSO), using an unpaired, two-tailed *t*-test. No significant differences were noted for any of the SC concentrations tested (one-way ANOVA, followed by Dunnett’s post-test). (**D**) Cell proliferation indicated by the sulforhodamine B assay, followed drug exposure up to 72 h. For each concentration, the mean ± SEM for at least three independent experiments is represented. No statistically significant differences were found between the different concentrations shown and the respective controls, for each time point (one-way ANOVA, followed by Dunnett’s post-test).

The toxicity of the SCs to NG108-15 cells was assessed using the MTT reduction, neutral red inclusion, and LDH release assays. According to the MTT reduction assay, THJ-2201 displayed higher cytotoxic potency than 5F-PB22 (Table 1, Figure 1A). THJ-2201 also exhibited a curve with greater steepness, meaning that for smaller increments in concentration, higher increases in cytotoxicity could be observed. Of note, significant differences to controls were only observed at high LOEC values for both SCs (Table 1).

Moreover, results depicted in Figure 1 also show that none of the tested SCs significantly decreased lysosomal degradation (neutral red inclusion, Figure 1B) or plasma membrane integrity (LDH release, Figure 1C) up to 25 µM. These results clearly indicate that at biologically relevant concentrations (<1 µM) none of the tested SCs is toxic to NG108-15 cells. In fact, it is largely accepted that the interaction of SCs with their receptors is concentration-dependent and that SC concentrations above 1 µM may activate secondary targets [18]. Therefore, from this point on we only used concentrations ≤1 µM to try to ascertain a possible cannabinoid receptor-mediated effect.

Cell proliferation represents a vital stage during neurodevelopment, and alterations on the regulation of this process can lead to severe consequences [19]. The potential role of the SCs on cell proliferation was assessed by the sulforhodamine (SRB) assay. As observed in Figure 1D, NG108-15 cells incubated with the SCs at concentrations between 1 pM and 1 μM showed proliferation curves similar to the control (cells growing in the absence of SCs), clearly evidencing the lack of effect of these substances on this parameter.

### 2.2. Mitochondrial Function Was Altered after Exposure to THJ-2201 and 5F-PB22

**Figure 2:**
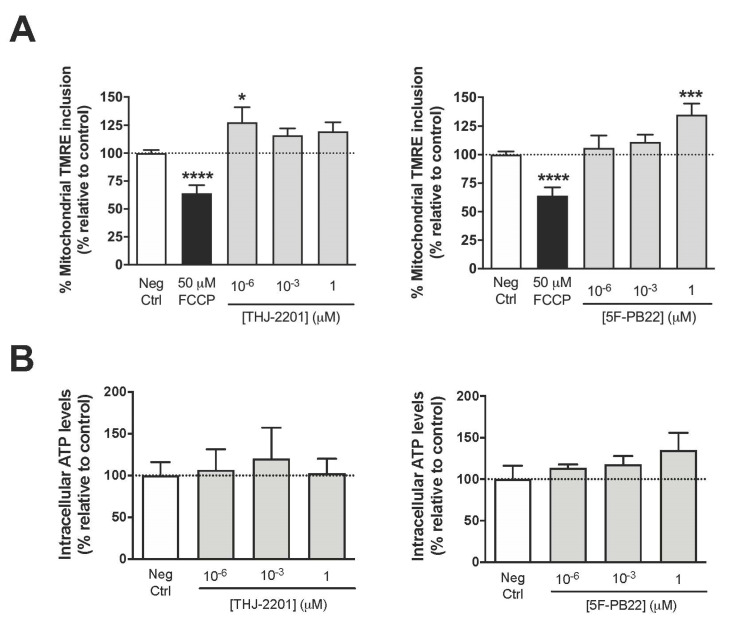
Assessment of mitochondrial parameters following NG108-15 cell exposure to 5F-PB22 and THJ-2201. NG108-15 cells were incubated for 24 h with the SCs at concentrations ranging from 1 pM to 1 μM. (**A**) Mitochondrial membrane potential, assessed through TMRE accumulation. 50 µM FCCP was used as positive control; (**B**) Intracellular ATP levels, measured using a luciferin-luciferase-based assay. Each bar represents the mean ± SEM, for at least six independent experiments, performed in duplicate. Results were normalized by the total amount of protein and expressed as the percentage relatively to the negative control (0.1% DMSO). * *p* < 0.05, *** *p* < 0.001, **** *p* < 0.0001, compared to the negative (vehicle-treated) control (one-way ANOVA, followed by Dunnett’s post-test).

Assessing mitochondrial membrane integrity is crucial to understand toxicological mechanisms. Figure 2A shows that after a 24 h-exposure, mitochondrial tetramethyl rhodamine ethyl ester (TMRE) inclusion increased in cells treated with 1 pM THJ-2201 (1.28-fold) or 1 μM 5F-PB22 (1.35-fold), indicating a SC-induced increase in mitochondrial membrane potential at these concentrations. Of note, these results contrasted the depolarization that occurred as a normal response to the mitochondrial proton uncoupler carbonyl cyanide-p-trifluoromethoxyphenylhydrazone (FCCP) [20].

Intracellular ATP levels depend on the balance between ATP production and consumption. In particular, ATP generation relies on ATP synthase, which uses the electrochemical proton gradient formed by the membrane potential and by the mitochondrial transmembrane proton concentration gradient to convert ADP and phosphate into ATP [21]. In addition, ATP may also be used in various cell mechanisms, from “housekeeping” events to the reversal of ion fluxes through postsynaptic receptors [22,23]. As shown in Figure 2B, none of the SCs tested, up to 1 μM, altered the net intracellular ATP levels in NG108-15 cells following 24 h-exposure.

### 2.3. Both THJ-2201 and 5F-PB22 Promoted Neuronal Differentiation

**Figure 3:**
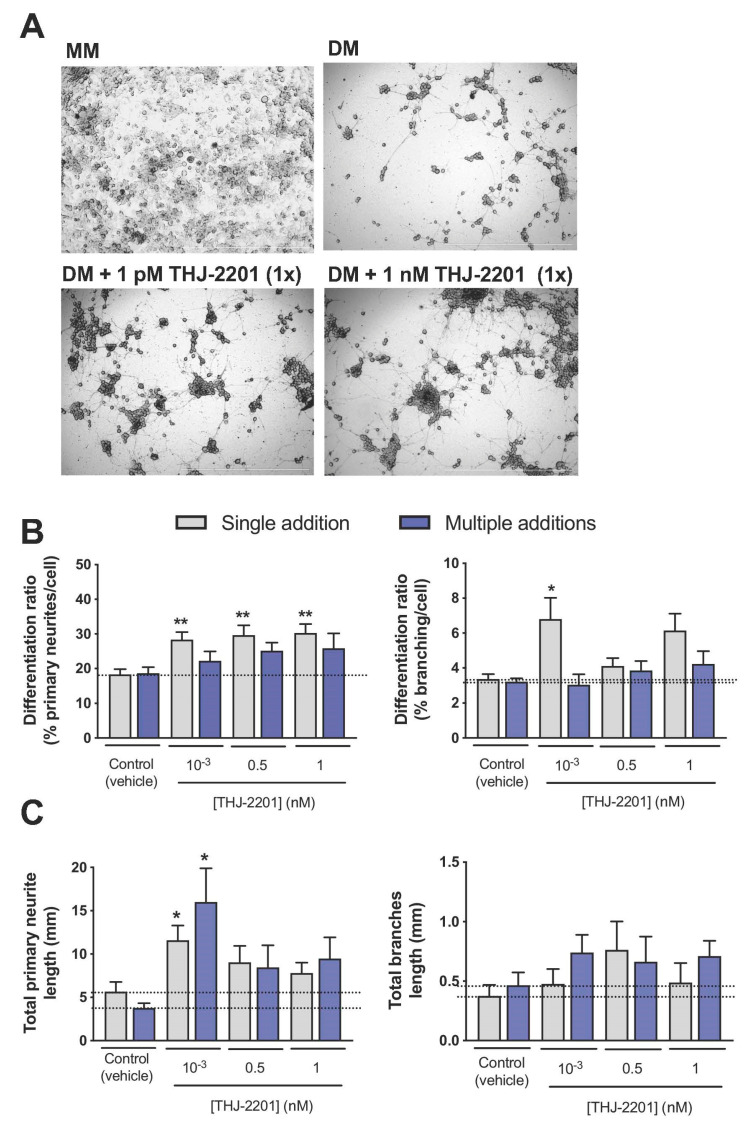
Effects of THJ-2201 on neuronal differentiation. Neuronal differentiation was induced by replacing maintenance medium (MM) by differentiation medium (DM) in the presence of the SC or the vehicle (0.1% DMSO). THJ-2201 was added to NG108-15 cells either once (single addition at day 0, in grey) or every 24 h up to 72 h (total of 3 additions, in purple). (**A**) Representative images of NG108-15 cells 72 h following induction of differentiation, and treated once with 0.1% DMSO (vehicle control) or THJ-2201, at 1 pM and 1 nM. Cells cultured in MM (in the absence of differentiation factors) continued to proliferate, preventing the analysis of neurite outgrowth. Scale bars correspond to 1000 μM; (**B**) differentiation ratios for primary neurites (left panel) and branches (right panel), calculated as the number of neurites longer than 20 µm divided by the total number of cells per well. (**C**) Total neurite length of primary neurites (left panel) and branches (right panel). Bars show the mean ± SEM, from at least five independent experiments, performed in duplicate. * *p* < 0.05, ** *p* < 0.01, compared to vehicle control (one-way ANOVA, followed by Dunnett’s post-test).

**Figure 4:**
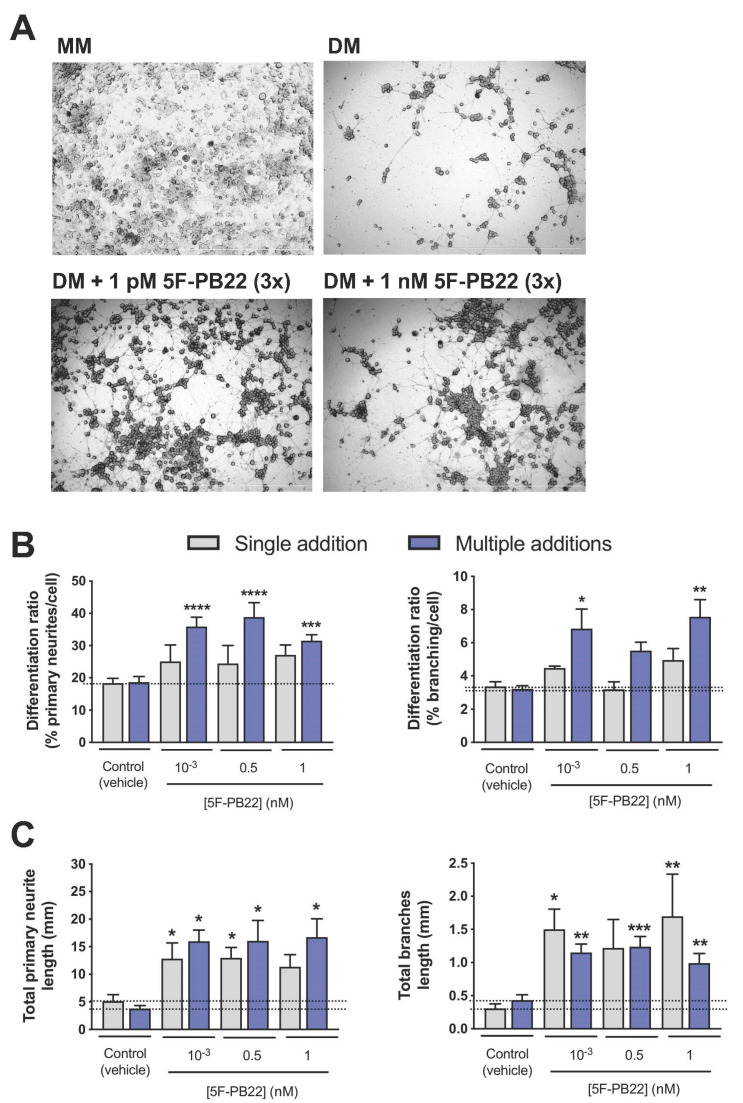
Effects of 5F-PB22 on neuronal differentiation. Neuronal differentiation was induced as reported in Materials and Methods. 5F-PB22 was added to NG108-15 cells either once (single addition at day 0, in grey) or every 24 h up to 72 h (total of three additions, in purple). (**A**) Representative images of NG108-15 cells 72 h following induction of differentiation, and treated once with 0.1% DMSO (vehicle control) or 5F-PB22, at 1 pM and 1 nM. Cells cultured in MM (in the absence of differentiation factors) continued to proliferate, preventing the analysis of neurite outgrowth. Scale bars correspond to 1000 μM; (**B**) differentiation ratios for primary neurites (left panel) and branches (right panel), calculated as the number of neurites longer than 20 µm divided by the total number of cells per well. (**C**) Total neurite length of primary neurites (left panel) and branches (right panel). Bars show the mean ± SEM, from at least five independent experiments, performed in duplicate. * *p* < 0.05, ** *p* < 0.01, *** *p* < 0.001, **** *p* < 0.0001, compared to vehicle control (one-way ANOVA, followed by Dunnett’s post-test).

**Figure 5:**
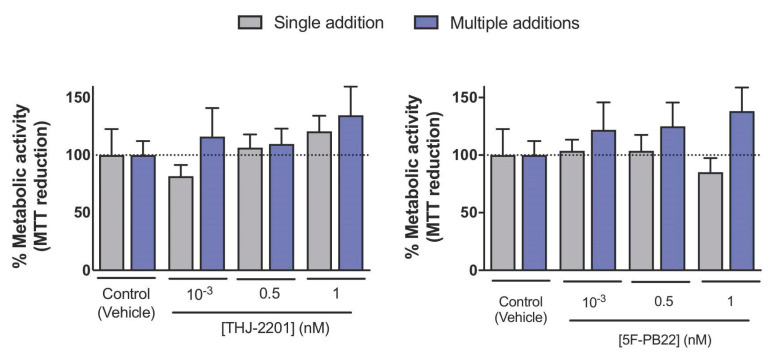
Metabolic activity of NG108-15 cells after the 3-day differentiation period, assessed by the MTT reduction assay. Neuronal differentiation was induced and NG108-15 cells were treated with 5F-PB22 or THJ-2201 as described in Materials and Methods. At the end of the differentiation process (day 3), cellular metabolic activity was determined according to the MTT reduction assay, similarly to undifferentiated cells (also described in Materials and Methods). Bars show the mean ± S.E.M for at least four independent experiments. No statistically significant changes were observed (one-way ANOVA, followed by Dunnett’s post-test).

Neuronal differentiation was assessed in NG108-15 cells by measuring the neurite outgrowth of cells exposed to both SC in Differentiation Medium (DM, DMEM supplemented with 1% FBS, forskolin and retinoic acid). As noted in Figure 3A and Figure 4A, cells cultured in DM exhibited clear signs of neurite outgrowth, contrasting cells in Maintenance Medium (MM), which continued to proliferate and reached a high cell density after three days in culture that prevented the proper analysis of neurite outgrowth. In this sense, all neuronal differentiation assays were performed in the presence of DM, thus facilitating the analysis of the effects of SCs on neuronal differentiation without the interference from other neurogenic processes such as cell proliferation. As also displayed in the representative images in Figure 3A and Figure 4A and further represented in Figure 3B and Figure 4B, neuronal differentiation ratios, especially considering primary neurites, increased near 2-fold following incubation with THJ-2201 (Figure 3) and 5F-PB22 (Figure 4) at concentrations below 1 nM, compared to the vehicle control. Branching was only significantly enhanced at 1 pM THJ-2201 or following exposure to 1 pM and 1 nM 5F-PB22 (Figure 3B and Figure 4B). Interestingly, these effects were observed following a single (THJ-2201) or repeated (5F-PB22) exposure, indicating that these two different SCs may require different dosages to induce the same effect. Of note, Figure 3C and Figure 4C evidence that in some conditions neurites formed in the presence of the SCs were also longer compared to the ones formed in DM treated with the vehicle alone. In particular, 1 pM THJ-2201 (but not the higher concentrations tested) increased total primary neurite length following both single (about 2-fold) and multiple (around 4.2-fold) treatments, although no significant differences were observed in terms of total length of branching (Figure 3C). On the other hand, 5F-PB22 induced a more pronounced effect on total neurite length, as all the concentrations tested increased the length of both primary and secondary/tertiary neurites, regardless of the exposure setting (Figure 4C).

Of note, the metabolic activity of differentiated cells was not affected by SC treatment, either following single or multiple additions, at concentrations below 1 nM, as depicted in Figure 5.

**Figure 6:**
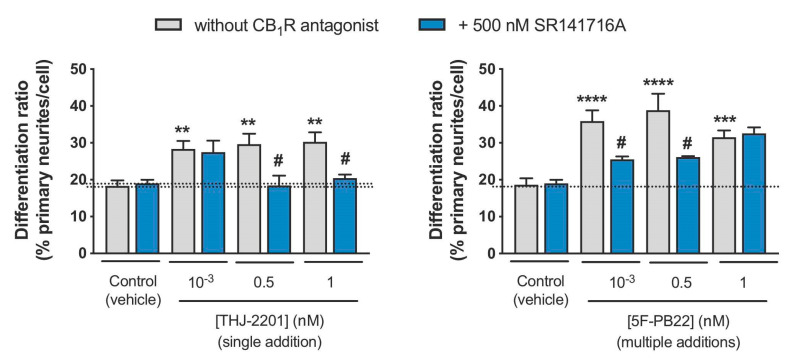
Assessment of CB_1_ receptor (CB_1_R) involvement in SC-mediated neuronal differentiation. Neuronal differentiation was induced as described in Materials and Methods and NG108-15 cells were treated with 5F-PB22 or THJ-2201 in the conditions that previously showed significant changes in primary neurite outgrowth (grey bars), following exposure 500 nM SR141716A, a selective CB_1_R antagonist (blue bars). Each bar represents the mean ± SEM, for at least five independent experiments, performed in duplicate. ** *p* < 0.01, *** *p* < 0.001, **** *p* < 0.0001, compared to vehicle control (one-way ANOVA, followed by Dunnett’s post-test). ^#^ *p* < 0.05, compared to the respective SC concentration in the absence of antagonist (one-way ANOVA, followed by Sidak’s multiple comparisons test).

The potential role of the CB_1_R in the enhanced neuronal differentiation mediated by THJ-2201 and 5F-PB22 was assessed by treating cells with a specific CB_1_R antagonist, SR141716, prior to (and along with) incubation with the SCs. As observed in Figure 6, the presence of SR141716 alone did not significantly modify the differentiation ratios. Moreover, the same figure evidences that the increase in differentiation ratios induced by both THJ-2201 and 5F-PB22 were significantly reduced following the blockade of CB_1_R activation. In particular, at the highest concentrations tested of THJ-2201 (500 pM and 1 nM), differentiation ratios in the presence of SR141716 were reduced back to basal levels. On the other hand, at the lowest concentrations tested of 5F-PB22 (1 pM and 500 pM), SR141716 decreased differentiation levels in about 60%. These results thus suggest that the regulation of neuronal differentiation by 5F-PB22 and THJ-2201 is mediated by the CB_1_R activation.

### 2.4. Expression of Neuronal Markers

Expression of neuronal markers was assessed by Western blot in differentiating NG108-15 cells, following exposure to SCs at the conditions that previously enhanced differentiation, that is, single exposure to THJ-2201 or repeated exposure to 5F-PB22. Four specific proteins were analyzed: β3-tubulin, a cytoskeleton protein mostly expressed in immature neurons [24]; synaptophysin, a synaptic vesicle protein that regulates vesicle endocytosis in neurons [25]; post-synaptic density protein 95 (PSD-95), a synaptic protein involved in the maturation of excitatory synapses [26]; and p73, which induces the expression of neurofilaments and neural cell adhesion molecule (N-CAM) [27,28].

**Figure 7:**
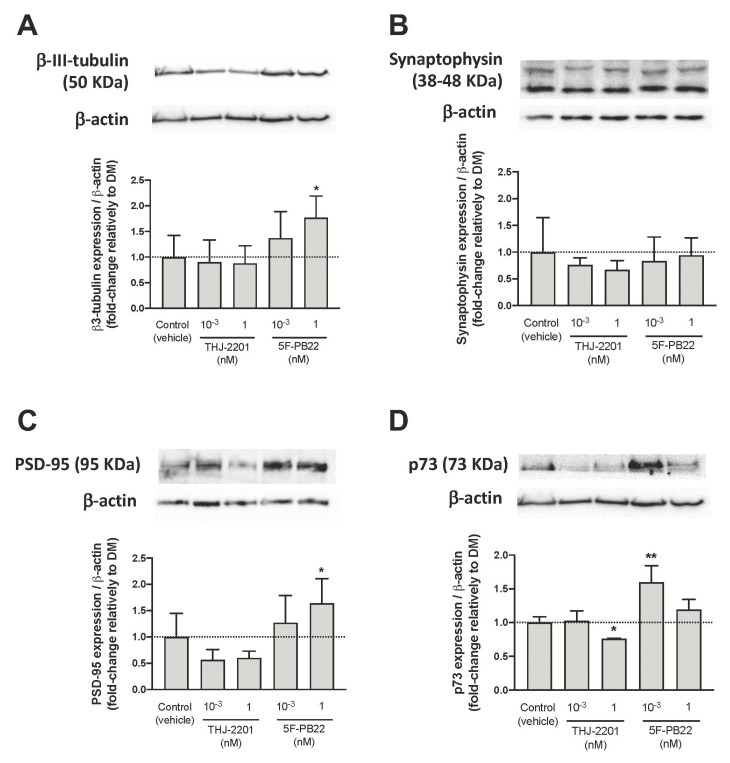
Expression of neuronal markers. Neuronal differentiation was performed according to the procedure described in Materials and Methods. After 72 h of differentiation and cell treatment with either a single addition of THJ-2201 (1 pM or 1 nM) or repeated (three) additions of 5F-PB22 (1 pM or 1 nM), the expression of (**A**) β3-tubulin (50 KDa), (**B**) synaptophysin (38–48 KDa), (**C**) PSD-95 (95 KDa), and (**D**) p73 (73 KDa) was assessed by Western blot. Representative protein bands and graphical representations of band intensities, expressed as the percentage of protein expression relatively to the vehicle-treated control, are shown for each protein. Their expression was normalized by the amount of β-actin per lane. Each bar represents the mean ± SD, for at least four independent experiments. * *p* < 0.05, ** *p* < 0.01, compared to control (vehicle), using a one-way ANOVA, followed by Dunnett’s post-test.

Figure 7 shows that 5F-PB22 significantly increased the expression of β3-tubulin and PSD-95 at 1 nM (Figure 7A,C, respectively), and of p73 at 1 pM (Figure 7D), compared to these markers’ expression in vehicle-treated cells. Surprisingly, despite having previously shown a significant increase in neurite outgrowth, treatment with THJ-2201 did not alter the expression of β3-tubulin (Figure 7A) or PSD-95 (Figure 7C), relatively to vehicle-treated cells. However, at 1 nM, THJ-2201 decreased p73 expression (Figure 7D). Interestingly, none of the tested SC conditions significantly affected the expression of synaptophysin (Figure 7B).

## 3. Discussion

The recreational use of SCs, which account for a high number of severe intoxications and deaths reported to the EMCDDA, remains very popular [29]. As the brain is a major target organ for SCs, we assessed the neurotoxicological profile of these substances, particularly focusing on elucidating whether they could impact neurogenic processes. Assessing the neurotoxic potential of SCs assumes extreme relevance considering their potential use by pregnant women and women of childbearing age. 

Our data show that two of the most commonly reported SCs, THJ-2201 and 5F-PB22, at biologically relevant concentrations (≤1 μM), i.e., SC concentrations that are broadly accepted to mainly trigger their primary mechanisms of action without eliciting significant secondary effects [18], did not significantly change cell viability parameters, including cell metabolic activity (MTT reduction), lysosomal integrity (neutral red inclusion), or plasma membrane integrity (LDH release). However, both SCs promoted the hyperpolarization of the mitochondrial membrane. We recently reported similar results in another cell model, as we showed that THJ-2201 disrupted the eCB-regulated mitochondrial function and triggered apoptotic cell death in human proximal tubule (HK-2) cells [30]. This mitochondrial involvement in SC-mediated toxicity is not surprising, since active CB1Rs have already been found in neuronal mitochondrial membranes [31]. Of note, targeting of mitochondria by SCs assumes high relevance considering the key role played by these organelles in several biological processes, namely during neurodevelopment [32]. No alterations were detected on intracellular ATP levels. However, it is not possible to draw any conclusion on the effect of the SCs on cellular energy production or metabolism, since the protocol we used only enables the assessment of net intracellular ATP levels. Still, considering that the total ATP levels were not changed, it is plausible to assume that SC-mediated neurotoxicity is not associated with the regulation of intracellular energy metabolism.

Most important, we found a clear modulation of in vitro neuronal differentiation following exposure of NG108-15 cells to THJ-2201 and 5F-PB22 at concentrations below 1 nM. Interestingly, repeated daily exposure to 5F-PB22 was required to produce a similar neurite outgrowth as the one induced by a single THJ-2201 dose. Although these two SCs produced similar differentiation ratios, 5F-PB22 promoted the formation of longer neurites and a higher ramification of the neurite network. Please note that NG108-15 cells differentiation in Maintenance Medium occurs concomitantly with proliferation, making any specific effect of the SCs on differentiation alone difficult to assess. By culturing the cells in Differentiation Medium, we removed (or at least reduced as much as possible) any likely interference from proliferation-related signaling events, thus specifically assessing the impact of SCs on neuronal differentiation.

In addition, regulation of both SC-mediated effects on neuronal differentiation appeared to be dependent on the activation of CB1R, as the inhibition of this receptor with a selective antagonist reverted the effects of both THJ-2201 and 5F-PB22. Similar effects have already been reported in the presence of other SCs. For example, Compagnucci et al. [33] described an increase in the differentiation of neural stem cells from mouse embryos after exposure to the synthetic CB1R-specific agonist arachidonyl-2′-chloroethylamide (ACEA). Similar to our findings, such results were reversed by cells’ co-treatment with AM251, an inverse CB1R agonist. However, data on the impact of SCs on neuronal differentiation have been contradictory, usually depending on several distinct factors, including the cell model used, tested SC and respective concentrations, or frequency of administration [9], suggesting that the role of SCs on neuronal differentiation may be likely related to the activation of different mechanisms. In fact, others have reported that CB1R activation by the non-selective CBR agonist WIN 55,212-2 inhibited new synapse formation in rat hippocampal neurons by preventing the formation of cyclic adenosine monophosphate (cAMP) [10]. Additionally, exposure of pregnant Wistar rats to the same SC (WIN 55,212-2) induced memory impairment in 40- and 80-day-old offspring, which was correlated with changes in hippocampal long-term potentiation and glutamate release [11]. Contrasting data on the effects of the synthetic cannabinoid HU-210 on differentiation have also been reported, as Jiang et al. [13] observed no differentiation of primary embryonic and adult hippocampal rat cells after chronic intraperitoneal injections of this SC to the animals, while Jordan et al. [34] found that HU-210 increased neurite outgrowth in Neuro-2A cells via CB1R-mediated activation of Rap1.

The ability of a given SC to trigger different cellular responses according to the concentrations tested or the frequency of administration could possibly explain the impact of single vs. multiple additions in the different differentiation ratios observed for THJ-2201 and 5F-PB22. Basavarajappa and Subanna [35] have suggested that the effects of acute SC exposure may be related to the stimulation of presynaptic CB1R-mediated signaling pathways and the inhibition of neurotransmitters release, whereas chronic exposure may trigger adaptive changes in the CB1R-related cascades of events. Moreover, Fay and Farrens [36] showed that ORG27569, a negative allosteric CB1R modulator, changed the conformational equilibrium of this receptor in a concentration-dependent way. The increased effects on differentiation ratios of a single THJ-2201 exposure compared to multiple additions, or the fact that only 1 pM THJ-2201 increased total neurite length despite all the concentrations tested having increased differentiation ratios, seems particularly intriguing. It is plausible that a higher exposure to THJ-2201, either related to higher concentrations or to repeated additions of this SC, may have resulted in the stabilization of different CB1R conformations. In addition, the possibility that such higher exposure to THJ-2201 may have caused neurite retraction to some extent, should not be fully discarded. In fact, considering that differentiation ratios represent the number of neurites per total cell number, neurite retraction due to high exposure to THJ-2201 could explain why all tested concentrations of THJ-2201 increased differentiation ratios (considering neurites as the extensions longer than 20 μm), but only 1 pM produced a significant increase in total neurite length.

Intriguingly, the CB1R antagonist did not seem to block the effects of 1 pM THJ-2201 and 1 nM 5F-PB22, which could be attributed to different hypothesis: (1) Cells exposure to different SC treatments (e.g., distinct concentrations and/or frequency of administration) may result in triggering pathways either dependent or independent of CB1R activation. In fact, Bambico et al. [37] have observed that low doses of the SC WIN55,212-2 exerted dose-dependent antidepressant-like effects in the rat forced-swim test and enhanced 5-HT neuronal activity in the dorsal raphe nucleus of SC-treated rats, via a CBR-dependent mechanism. However, high doses of the same SC proved ineffective in the forced-swim test and decreased 5-HT neuronal activity by a CBR-independent mechanism; (2) different SC concentrations, in combination with SR141716A, may have induced biased signaling towards different CB1R subunits. Similar data has been observed by Laprairie et al. [38], who reported that the combination of THC (a partial CBR agonist) and cannabidiol (a CBR antagonist) targeted biased signaling toward Gα_s_ compared to Gα_i/o_, although signaling was biased toward Gα_i/o_ compared to β-arrestin-1, Gα_q_ and Gβγ.

Neurogenesis requires a well-orchestrated series of complex regulatory events [39]. In this sense, we further analyzed how these SCs affected the expression of different proteins involved in neuronal differentiation and maturation, including β3-tubulin, synaptophysin, PSD-95, and p73. β3-tubulin is a neuron-specific cytoskeletal protein, markedly expressed during fetal and post-natal development, thus being mostly present in immature neurons [40,41]. Synaptophysin is the most abundant synaptic vesicle membrane protein, playing a key role in regulating the kinetics of synaptic vesicle endocytosis in neurons, and being often used as marker for pre-synaptic terminals in mature neurons [42]. PSD-95 is essential for synaptic maturation and plasticity, being highly present in the excitatory post-synaptic density, promoting the clustering of proteins like glutamate receptors on the post-synaptic membrane and coupling them with downstream signaling molecules [39]. p73 is a pleiotropic protein of the p53 family that plays an important role during differentiation. An increase in p73 levels has been associated with enhanced neurite outgrowth and increased levels of biochemical markers related to neuronal differentiation (e.g., expression of neurofilaments and N-CAM) following retinoic acid-induced differentiation [27].

We observed that β3-tubulin, PSD-95, and p73 were overexpressed following exposure to repeated treatments of 5F-PB22. These results are in line with the data regarding neurite outgrowth and further support the evidence that 5F-PB22 promotes neuronal differentiation. In fact, based on these results, a mixed population of differentiating and already differentiated neurons seems to be present after 72 h of multiple exposure to this drug, as indicated by the higher β3-tubulin (immature neurons), and PSD-95 and p73 (mature neurons) expression. Interestingly, none of the SCs tested altered the expression of synaptophysin, suggesting that these substances do not affect synaptic vesicle endocytosis, thus likely not interfering with synaptic transmission during neuronal differentiation.

Surprisingly, in addition to the absence of changes in synaptophysin expression, we found no significant modification of β3-tubulin or PSD-95 expression in the THJ-2201 conditions that had previously shown enhanced neurite outgrowth. In fact, 1 nM THJ-2201 reduced p73 expression. While seemingly contrasting the higher neurite outgrowth, these data could anticipate an impairment on neuronal maturation. Of note, p73-deficient mice have been reported to show marked neuronal defects, in particular in the CA1-CA3 pyramidal cell layers of the hippocampus and in the dentate gyrus, besides displaying reduced cortical thickness as a consequence of the loss of mature cortical neurons [28]. Moreover, considering that β3-tubulin is mostly expressed in the early periods of neuronal differentiation [43] and that the other markers tested are usually found in mature neurons [39], it may be reasonable to expect that cells differentiated in the presence of THJ-2201 may have not reached a mature state.

Interestingly, Ly and co-workers [44] have shown that other psychoactive substances such as lysergic acid diethylamide (LSD), N-dimethyltryptamine (DMT), 2,5-dimethoxy-4-iodoamphetamine (DOI), or ketamine increase neurogenesis and synaptogenesis with a subsequent increase in neuroplasticity. Of note, higher neuroplasticity enables the brain to better respond to changes in the surrounding environment, having been associated with a reduction of both anxiety and depression-like behaviors [45]. In this context, our findings could possibly anticipate a beneficial effect on neuronal function for the SCs herein tested. However, since in homeostatic conditions a neuronal stem cell only commits to differentiation when required and within the correct time frame [46,47], it may also be reasonable to expect that an unsolicited increase in neuronal differentiation (e.g., induced by an exogenous factor, like SC administration), may result in central nervous system (CNS) malformations. In fact, fetal exposure to 2.0 mg/kg of the SC CP-55,940 has been reported to induce CNS abnormalities like exencephaly, holoprosencephaly, or cortical dysplasia [48].

We also observed that THJ-2201 and 5F-PB22 did not affect neuronal cell proliferation. Of note, Ferreira and co-workers [49] have previously reported that HU-308 (a synthetic CB2R agonist) and WIN 55,212-2, both at 1 μM, did not promote significant changes in the number of BrdU-positive cells compared to control cultures. However, Jacobsson et al. found that WIN55,212-2 positively modulated neuronal proliferation through CB1R activation in C57BL/6 mice [50]. In addition, HU-210 was also reported to induce proliferation of rat cultured embryonic neural stem and progenitor cells after 48 h [13]. Altogether, the different effects observed for SCs on neuronal proliferation are likely associated with the SC tested (e.g., considering different molecular structures or concentrations used), the time of exposure, dosage regimen (i.e., acute vs chronic) the different in vitro/in vivo models employed, or even the brain region analyzed, as noted by Ferreira et al. [49]. In fact, it should be noted that while we used a homogeneous cell line in terms of cell identity, other studies were performed in models comprising higher cell heterogeneity, such as the case of animal assays, or primary embryonic neural stem and neural progenitor cell cultures [10,11,13,14], thus possibly explaining the contrasting outcomes.

Overall, we report firsthand the CB1R-mediated enhancement of neuronal differentiation of NG108-15 cells by THJ-2201 and 5F-PB22 at biologically relevant concentrations, noting that these two SCs required different dosage regimens to attain similar differentiation ratios. Of note, exposure of NG108-15 cells to 5F-PB22 resulted in longer neurites and higher number of branches. Moreover, while different proteins specifically involved in neuronal differentiation and maturation, namely β3-tubulin, p73 or PSD-95 were found overexpressed in response to a repeated 5F-PB22 treatment, THJ-2201, surprisingly, only reduced p73 expression, suggesting the involvement of distinct mechanisms in the modulation of neuronal differentiation promoted by these two SCs. 

Nevertheless, the effects of both tested SCs on neuronal differentiation depended on the activation of CB1R, suggesting that each of the SCs may induce a different response from these receptors, which requires further clarification. In fact, the involvement of CB1R in the activation of distinct signaling cascades, including the inhibition of the adenylyl cyclase (AC)-cyclic AMP-protein kinase A (PKA) pathway, activation of mitogen-activated protein kinase (MAPK) cascades, or the regulation of ion flux via inhibition of voltage-sensitive Ca2+ channels (VSCC) has already been described [9]. Moreover, it has been reported that an agonist binding to a given G protein-coupled receptor, as is the case of CBRs, can stabilize distinct receptor conformations and subsequently activate different signaling cascades that may favor one signaling pathway over the other. Activation of the Gα_i/o_ heterotrimer over β-arrestin-1 prompts the release of the Gβγ subunit, which inhibits voltage-dependent calcium channels and activates G protein-gated inwardly rectifying potassium channels (GIRK). The Gα_i/o_ subunit then inhibits adenyl cyclase, stimulating the phosphorylation and early activation of ERK1/2. On the other hand, the activation of β-arrestin-1 triggers the late activation of ERK1/2 [51]. For example, two similar SCs, JWH-018 and JWH-081, have been reported to act on CB1R. However, they have been also described as activating distinct CB1R-mediated signaling events. JWH-018 is known to decrease pERK1/2 expression [52], whereas JWH-081 does not affect pERK1/2, rather impairing pCaMKIV and pCREB levels, which are linked to the expression of the activity-regulated cytoskeleton-associated (Arc) protein, a protein associated with the regulation of neuronal plasticity [53].

Of note, none of the SCs tested significantly affected most of the toxicity-related parameters at biologically relevant concentrations. However, they both induced the hyperpolarization of mitochondrial membrane, suggesting a possible regulatory action of mitochondrial function. Although these data support the potential of SCs to affect neuronal development, especially considering their potentially adverse outcomes to differentiating neurons, further research is required to fully understand the underlying mechanisms involved in the modulation of neurodevelopment.

## 4. Materials and Methods

### 4.1. Chemicals

**Figure 8:**
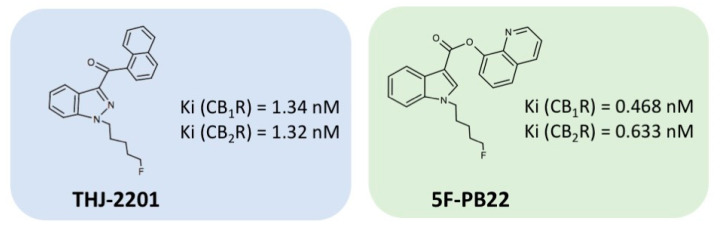
Structures of THJ-2201 and 5F-PB22 and respective binding affinities to the cannabinoid receptors (CBRs) type 1 and 2, according to Hess et al. [54].

5F-PB22 (also known as 5F-QUPIC or quinolin-8-yl-1-pentylfluoro-1H-indazole-3-carboxylate) and THJ-2201 ([1-(5-fluoropentyl)-1H-indazol-3-yl](1-naphthyl)methanone) were kindly supplied by Dr Ana Santos Carvalho (Centre for Neurosciences and Cell Biology, University of Coimbra, Portugal). 5F-PB22 is the terminally fluorinated analogue of PB-22, comprising a quinolone substructure connected by an easily biodegradable ester linkage to a substituted indole ring [54]. THJ-2201 is a structural analogue of AM-2201, with an indazole group replacing the central indole ring (Figure 8). Confirmation of its identity and relative purity (96.6%) determined by Gas Chromatography coupled to Mass Spectrometry (GC-MS) was previously reported [29]. SR141716A, a specific antagonist for the CB1R, was purchased from Tocris Bioscience (Bristol, UK). Heat-inactivated fetal bovine serum (FBS), antibiotic (10 000 U/mL penicillin, 10 000 μg/mL streptomycin), 0.25% trypsin/EDTA, phosphate buffered saline (PBS), and Hank’s balanced salt solution (HBSS) were acquired from Gibco Laboratories (Lenexa, KS, USA). All other reagents used in this work were purchased from Sigma Aldrich (St. Louis, MO, USA), unless stated otherwise.

Stock solutions of the SCs and SR141716A were prepared in dimethyl sulfoxide (DMSO). These stock solutions were sequentially diluted in HBSS before cell exposure to attain a final DMSO concentration below 0.1%. This concentration has been previously described as being non-toxic to NG108-15 cells and below the minimum threshold required to promote NG108-15 differentiation, at low serum conditions [55].

### 4.2. Cell Culture

The mouse neuroblastoma/rat glioma hybrid NG108-15 cell line was acquired from the European Collection of Authenticated Cell Cultures (ECACC, Salisbury, UK) and was routinely cultured in 75 cm^2^ flasks with Dulbecco’s Modified Eagle’s Medium (DMEM), supplemented with 10% (*v*/*v*) heat-inactivated FBS and 100 U/mL penicillin and 100 μg/mL streptomycin. The cells were maintained at 37 °C in a humidified atmosphere containing 5% CO_2_. Once the plates reached 70–80% confluence, the cells were sub-cultured by trypsinization with a 0.25% trypsin/EDTA solution. Differentiated NG108-15 cells have been reported to allow a prompt neurotoxicological evaluation [55] and are preferred over primary cells as a model for neuronal differentiation studies, since they: (1) allow assessing the adhesive and morphological alterations occurring during that process; (2) grow more rapidly than primary cultures in culture medium; (3) show a homogeneous cell type identity; and (4) display synchronous differentiation in culture dishes. They endogenously express CB_1_R, the CBR mainly expressed in the brain, representing a further advantage to particularly study SCs neurotoxicity [15,56].

### 4.3. Cell Viability

#### 4.3.1. MTT Reduction Assay

The cytotoxicity of 5F-PB22 and THJ-2201 was evaluated through the 3-(4,5-dimethylthiazol-2-yl)-2,5-diphenyltetrazolium bromide (MTT) reduction assay, as described before [57]. The MTT assay indirectly evaluates cell metabolic activity through the assessment of the activity of cell reductases that convert yellow soluble MTT into purple insoluble formazans. Briefly, non-differentiated NG108-15 cells seeded onto 96-well plates at a density of 2 × 10^4^ cells/well, were exposed to each drug for 24 h. The concentration range varied from 1.5 × 10^−7^ to 2 mM to enable complete concentration-response curves. Vehicle-treated (0.1% DMSO) and positive (1% Triton X-100) controls were also included. After 24-h-incubation, the cell culture medium was removed and 100 μL of 1 mg/mL MTT solution were added to each well. The cells were further incubated at 37 °C, in a 5% CO_2_ atmosphere, for 1 h. Then, the MTT solution was aspirated and the intracellular formazan crystals were dissolved with 100 μL of DMSO. The plate was shaken for 15 min and protected from light. The absorbance of the colored solution was measured at 550 nm, directly in the plate, using a BioTek Synergy^TM^ HT (BioTek Instruments, Inc.) multi-well plate reader. Data were normalized to positive and vehicle-treated controls, and graphically presented as the percentage of reduced MTT relative to negative controls.

#### 4.3.2. Neutral Red Inclusion

Neutral red dye permeates the plasma membranes of viable cells and accumulates in lysosomes via non-ionic diffusion. This lysosomal uptake occurs due to the presence of a proton gradient between lysosomes and the cytoplasm. The disruption of this gradient, which may result from cell damage or death, hampers neutral red retention in the lysosomes, and the dye is eventually removed during the washing steps of this protocol. As a result, it is possible to establish a correlation between the neutral red signal and cell viability [58]. Neutral red uptake was determined as previously described [59]. Cells were seeded at 1.5 × 104 cells/well in 96-well plates and exposed to the SCs at a concentration range of 1 pM–25 µM. Vehicle-treated (0.1% DMSO) and positive (1% Triton X-100) controls were also tested. Following 24 h of exposure to the SCs, cell culture medium was replaced by 100 μL of a 50 μg/mL neutral red solution prepared in fresh medium and the cells were further incubated for 1 h, at 37 °C, 5% CO_2_. Cells were then lysed using a 50% ethanol/1% glacial acetic acid solution to extract the dye retained in the lysosomes. Complete dye dissolution was attained by placing the plates on an orbital shaker for 15 min and absorbance was then read at 540 nm in a Bio-Tek PowerWaveX (Bio-Tek, Winooski, VT, USA) microplate reader. Results were then expressed as the percentage of neutral red uptake by lysosomes relative to the vehicle-treated control.

#### 4.3.3. Lactate Dehydrogenase (LDH) Release

Cell membrane integrity was determined by measuring the release of lactate dehydrogenase (LDH), a membrane leakage marker, into the extracellular medium, as previously described [60]. NG108-15 cells were seeded in 96-well plates at 1.5 × 10^4^ cells/well and exposed to the SCs at concentrations ranging between 1 pM and 25 µM. Vehicle-treated (0.1% DMSO) and positive (1% Triton X-100) controls were also tested. After a 24 h-incubation, cell culture medium was collected to quantify the extracellular LDH. Cells were then lysed with 10 mM HEPES (pH 7.4) containing 0.01% Triton X-100 and frozen at −20 °C for later quantification of intracellular LDH. Debris from both extra- and intracellular samples were removed by centrifugation at 9400× *g*, for 10 min in a Heraeus Biofuge Fresco centrifuge (Hanau, Germany). The enzyme activity was assessed spectrophotometrically in a Bio-Tek PowerWaveX (Bio-Tek, Winooski, VT, USA) microplate reader at 340 nm, by following the rate of conversion of 0.28 mM reduced nicotinamide adenine nucleotide (NADH) into oxidized nicotinamide adenine nucleotide (NAD^+^), for 5 min, using 0.32 mM pyruvate (prepared in phosphate buffer, pH 7.4) as substrate. Results were then expressed as the percentage of LDH released relatively to the total (intracellular + extracellular) LDH activity.

### 4.4. Sulforhodamine B (SRB) Assay

The effects of SCs on cell proliferation were assessed by measuring the total cellular protein content by the SRB protein staining assay, as previously described [61], with slight modifications. SRB binds to basic amino acids of cellular proteins and its colorimetric evaluation provides an estimate of total protein mass, which correlates to cell number. Evaluation of SRB staining over time provides a reliable indication of cell proliferation [62]. NG108-15 cells were seeded at 3.5 × 10^4^ cells/well in 24-well plates and incubated in the presence of the SCs at concentrations between 1 pM and 1 nM during 24, 48, and 72 h. At each time point, cell culture medium was discarded, cells were fixated in 1 mL of a 1% acetic acid solution in methanol and the plates stored at −80 °C for later (up to a week) SRB staining quantification. After thawing, the fixation solution was removed and the plates were allowed to dry at 37 °C for 15 min. Then, 250 µL of a 0.5% SRB solution in 1% acetic acid was added to each well and the plates further incubated at 37 °C for 1.5 h, protected from light. At the end of this period, the plates were thoroughly washed with 1% acetic acid solution to remove the excess of SRB solution. The plates were allowed to dry in an oven at 37 °C and 1 mL of a 10 mM Tris solution was then added to each well to dissolve the bound SRB. Two hundred microliters were transferred from each well onto a 96-well plate and the absorbance read at 540 nm in a Bio-Tek PowerWaveX (Bio-Tek, Winooski, VT, USA) microplate reader, using a 10 mM Tris solution as blank. Results were expressed as the percentage of SRB binding relatively to the vehicle-treated cells at 0 h, which was considered as 100% cell viability.

### 4.5. Mitochondrial Function

Cells’ mitochondrial integrity was assessed by measuring the electrophoretical accumulation of the positively-charged TMRE dye in active mitochondria. TMRE accumulates in mitochondria proportionally to the mitochondrial membrane potential (ΔΨm), due to their relative negative charge [63]. The TMRE assay was prepared by seeding the cells at an 8 × 10^4^ cells/mL density in 96-well plates, as previously described [60]. Cells were incubated for 24 h with the SCs at concentrations ranging from 1 pM to 1 µM. After this period, cell culture medium was removed, the wells were washed twice with HBSS and the cells were incubated with 100 µL of a 2 µM TMRE solution (prepared in cell culture medium) for 30 min, at 37 °C, 5% CO_2_. The TMRE solution was then discarded by aspiration and the cells rinsed twice with 0.2% bovine serum albumin (BSA) in HBSS. Fluorescence was read in a BioTek Synergy^TM^ HT (BioTek Instruments, Inc.) microplate reader at 544 nm excitation/590 nm emission. Fifty micromolar carbonyl cyanide-p-trifluoromethoxyphenylhydrazone (FCCP) was used to confirm that the assay was properly functioning. FCCP is an ionophore that uncouples oxidative phosphorylation, eliminating mitochondrial membrane potential and reducing TMRE staining. Results were normalized by total protein content per well, as assessed by the SRB assay, and then expressed as the percentage of mitochondrial TMRE inclusion relative to the negative control.

### 4.6. Intracellular ATP Levels

Intracellular net levels of adenosine 5′-trifosfate (ATP) were determined according to a previously described procedure [60]. This method is based on the emission of bioluminescence derived from the luciferase-catalyzed reaction between luciferin and intracellular ATP. The intensity of bioluminescence is proportional to the ATP levels in the sample [64]. Briefly, cells were seeded at 1 × 10^5^ cells/well in 24-well plates and incubated with SCs for 24 h (at a concentration range of 1 pM–1 nM). Following cell exposure to the SCs, cells were washed with HBSS and precipitated with 200 µL of 5% perchloric acid and further incubated for 20 min at 4 °C. Cells were then scrapped and collected into 1.5 mL tubes, which were then centrifuged at 6000× *g* for 5 min at 4 °C. The supernatants were collected into new 1.5 mL tubes and neutralized with 400 µL of 0.76 M KHCO_3_, while the pellets were resuspended in 0.3 M NaOH and used to determine the total amount of protein through the Lowry method. The solutions containing the neutralized supernatants were mixed by vortexing and further centrifuged for 1 min at 9400× *g*, at 4 °C. The reaction was then initiated by mixing 75 µL of each supernatant with 75 µL of luciferin-luciferase reagent at a final luciferase concentration of 3,000,000 U/mL in a 50 mM glycine, 10 mM MgSO_4_, 1 mM Tris, 0.55 mM EDTA and 1% BSA buffer, pH 7.6. ATP levels were determined by interpolation from an ATP standard calibration curve, normalized by the total protein amount, and expressed as the percentage compared to the vehicle-treated control.

### 4.7. Neuronal Differentiation

Differentiation of NG108-15 cells was induced according to a previously described procedure [65], with slight modifications. Briefly, cells were plated in 96-well plates at a density of 1.5 × 104 cells/mL and allowed to adhere overnight. Differentiation into mature neurons was then induced by replacing the complete cell culture medium (hereafter referred to as maintenance medium, MM) by DMEM supplemented with 1% FBS, 30 µM forskolin, and 10 µM retinoic acid to promote differentiation (hereafter referred to as differentiation medium, DM). SCs were added at concentrations ranging from 1 pM to 1 nM either once, in acute (single addition, at 0 h, immediately after replacement of MM by DM) or repeated (every 24 h up to 72 h, in a total of three additions) exposure settings. To analyze the role of CB1R on SC-mediated neuronal differentiation, 500 nM SR141716A, a specific CB1R antagonist, was added 20 min prior to SCs exposure, according to the procedure described by Silva et al. [60]. A vehicle-treated control (cells maintained in DM, in the presence of 0.1% DMSO) was also included. To check whether the differentiation was running properly, a condition in which NG108-15 cells were maintained in MM after day 0 was also included. Of note, cells in this condition continued to proliferate, showing none or reduced differentiation. After 72 h of incubation (day 3), neurite outgrowth in each condition was imaged using phase contrast with the Lionheart™ FX Automated Microscope (Bio-Tek, Winooski, VT, USA). Determination of neurite outgrowth was performed by measuring the neurite length and total cell number using the Neurite Tracer plugin or the multi-point tool, respectively, within the ImageJ open source image processing software (ImageJ 2.0.0 National Institutes of Health, Bethesda, MD, USA). Differentiation ratios were calculated as the number of neurites per total number of cells per well. We considered neurites as every outgrowth extending longer than 20 µm from the soma, in accordance with Campanha et al. [15].

### 4.8. Total Protein Extraction

The expression of specific proteins associated with neuronal differentiation was analyzed by Western blot in total protein extracts of NG108-15 cells differentiated in the presence or absence of the SCs. Cell differentiation and SC treatment were performed at a density of 3.0 × 10^5^ cells/well in 6-well plates, in the same conditions described for the differentiation assay (Section 4.7). Following the 72 h-incubation, cell culture medium was collected, and cells were scrapped in the presence of HBSS and collected into 15 mL tubes. The wells were further rinsed with 1 mL HBSS and the suspension collected into the same tubes. Cell suspensions were centrifuged at 1000× *g* for 5 min and supernatants were discarded. The cells were resuspended in 150 µL of collecting buffer (20 mM HEPES, 250 mM sucrose, 10 mM KCl, 2 mM MgCl_2_, 1 mM EDTA, pH 7.5) supplemented with 2 mM dithiothreitol (DTT) and 100 µM phenylmethylsulfonyl fluoride. The pellets were then disrupted by sonication with three pulses of 30 s intercalated with 30 s on ice. Quantification of total protein in the cell extracts was determined using the Bio-Rad Detergent Compatible (DC) protein assay (Bio-Rad, Hercules, CA, USA), according to the manufacturer’s instructions. The samples were then stored at 80 °C until used.

### 4.9. Western-Blot Analysis

Protein expression following SC-treatment was determined by Western blot on total cell extracts, as previously reported [60]. Equal amounts of samples (containing 50 µg of protein) were denatured at 90 °C for 3 min in sample loading buffer (0.25 M Tris-HCl, 50% glycerol, 10% sodium dodecyl sulphate (SDS), 0.2 M DTT and 0.001% bromophenol blue). Samples were separated by electrophoresis in 10–15% SDS-polyacrylamide gels and transferred to polyvinylidene fluoride membranes (GE Healthcare, Pittsburgh, PA, USA). Membranes were blocked in 5% skimmed milk prepared in 0.05% Tween 20 in phosphate buffered saline (TPBS, pH 7.4) for 2 h at room temperature, in an orbital shaker. The membranes were then washed three times, for 10 min each, with TPBS and further incubated overnight, at 4 °C, with the following primary antibodies: mouse anti-β3-Tubulin (1:250, Santa Cruz Biotechnologies, Santa Cruz, CA, USA), mouse anti-PSD95 (1:250, Santa Cruz Biotechnologies, Santa Cruz, CA, USA), mouse anti-synaptophysin (1:250, Santa Cruz Biotechnologies, Santa Cruz, CA, USA), and mouse anti-p73 (1:250, Santa Cruz Biotechnologies, Santa Cruz, CA, USA). Blots were also probed for mouse anti-β-actin (1:4000, Sigma-Aldrich, St Louis, MO, USA) to ascertain equal sample loading. Primary antibodies were diluted in 1% BSA prepared in TPBS, supplemented with 0.05% sodium azide. The membranes were further washed in TPBS (three consecutive washes of 5, 10 and 15 min) and further incubated with horseradish peroxidase-conjugated anti-mouse immunoglobulin (IgG, 1:2500, GE Healthcare, PA, USA) diluted in 1% BSA prepared in TPBS for 1 h, at room temperature under stirring. The membranes were washed in TPBS (three consecutive washes of 5, 10 and 15 min) and protein bands were detected by incubating the membranes in Clarity Western ECL Substrate (Bio-Rad, Hercules, CA, USA) for 5 min. The membranes were then imaged using the molecular imager ChemiDoc^TM^ XRS (Bio-Rad, Hercules, CA, USA). Band intensities in each lane were quantified using the Bio-Rad Image Lab (Version 5.1, Build 7, Bio-Rad, Hercules, CA, USA) and normalized against the intensities of the endogenous control β-actin. Results were expressed as the fold-change relative to the control (cells treated with 0.1% DMSO).

### 4.10. Statistical Analysis

Statistical analysis was performed using GraphPad Prism 8 software (GraphPad Software, La Jolla, CA, USA). The normalized MTT data were fitted to the dosimetric Logit model, which was chosen based on a statistical goodness-of-fit principle [66]: *y* = *θ*max/{1 + exp [−*θ*1 − *θ*2 × log (*x*)]}, where *θ*max is the maximal observed effect, *x* is the concentration of test drug, *θ*1 is the parameter for location and *θ*2 is the slope parameter. The lowest observed effect concentration (LOEC) and the no observed effect concentration (NOEC) for each drug were calculated by testing a trend in concentration effects against controls by applying hypothesis-testing procedures (unpaired *t* tests) [57], which allowed the estimation of LOEC. Consequently, the next lower test concentration could be assigned as the NOEC. For other assays, analysis of the normality of each distribution was assessed using the Anderson–Darling, D’Agostino–Pearson and Shapiro–Wilk normality tests, and taking into account the acceptability of skewness and kurtosis values. Based on the normality results, one-way ANOVA, followed by a Dunnett’s post-hoc test or Sidak’s multiple comparisons test (to compare the effects of SCs in presence vs absence of CB1R antagonist), or unpaired two-tailed *t*-test (to compare positive vs vehicle controls) were performed, as appropriate. The number of independent experiments, as well as the number of replicates assayed, if any, are detailed in the figures’ legends.

## Figures and Tables

**Table 1:** Estimated parameters for the Logit model (best-fit regression) of 5F-PB22 and THJ-2201 in the 3-(4,5-dimethylthiazol-2-yl)-2,5-diphenyltetrazolium (MTT) reduction assay, after 24 h-incubation of non-differentiated NG108-15 cells, at 37 °C.

Drug	Parameters for the Logit Regression Model			EC_50_ (mM)	NOEC (μM)	LOEC (μM)
	θ_1_^a^	θ_2_^b^	θ_max_^c^			
**5F-PB22**	−0.68	1.54	141.80	1.12	5.86	11.72
**THJ-2201**	1.03	3.86	85.78	0.66	78.12	150

EC_50_, concentration of drug that induces half-maximal response, i.e., 50% effect in the assay. NOEC, no observed effect concentration. LOEC, lower observed effect concentration. ^a^ Location parameter. ^b^ Slope parameter. ^c^ Maximal effect, expressed as % cell death (data scaled between 0 and 100% cell death, corresponding to negative controls and positive controls, respectively).
